# Correction: Hayashi et al. More Tolerant Reconstructed Networks Using Self-Healing against Attacks in Saving Resource. *Entropy* 2021, *23*, 102

**DOI:** 10.3390/e24111530

**Published:** 2022-10-26

**Authors:** Yukio Hayashi, Atsushi Tanaka, Jun Matsukubo

**Affiliations:** 1 Graduate School of Advanced Science and Technology/Division of Transdisciplinary Sciences, Japan Advanced Institute of Science and Technology, Nomi 923-1292, Japan; 2 Graduate School of Science and Engineering, Yamagata University, Yonezawa 992-8510, Japan; 3 Department of Creative Engineering, National Institute of Technology Kitakyushu College, Kitakyushu 802-0985, Japan

The authors wish to make the following correction to this paper [1]. There was a careless mistake in Figure 5 (calculated by using link-based data instead of the usual node-based data for this *k_avg_*(*q*) alone): the virtual scale was doubled. However, because of the comparison with the methods represented by colored lines, replacing this figure does not affect to the substantive meaning of the results obtained, including our conclusion, or anything other than the revised sentences.

## Figures and Tables

**Figure 5:**
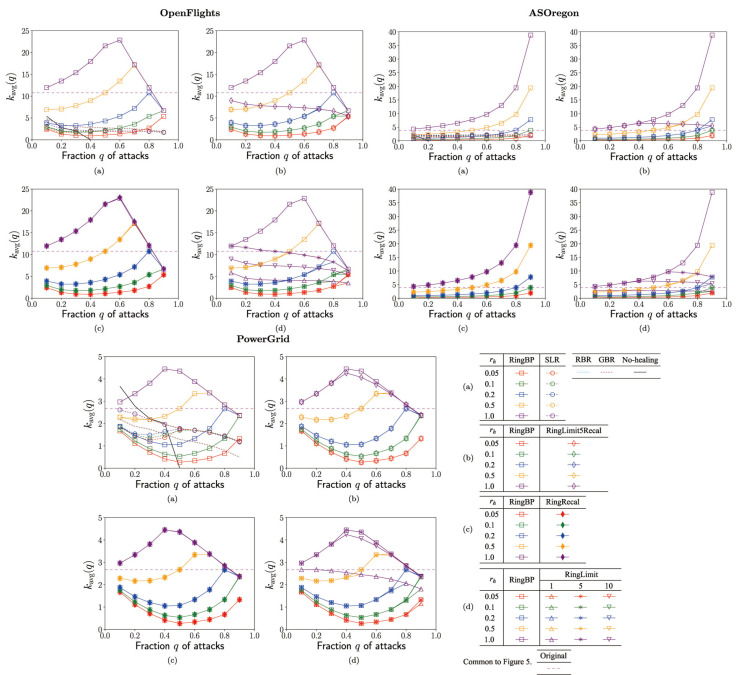
Average degree *k_avg_*(*q*) in the surviving *N_q_* nodes vs. fraction *q* of attacks for the rate *r_h_* in rewirings. (**a**) SLR, RBR, GBR, RingBP, (**b**) RingBP, RingLimit5Recal, (**c**) RingBP, RingRecal, and (**d**) RingBP, RingLimit1,5,10 methods.
